# PRO B: evaluating the effect of an alarm-based patient-reported outcome monitoring compared with usual care in metastatic breast cancer patients—study protocol for a randomised controlled trial

**DOI:** 10.1186/s13063-021-05642-6

**Published:** 2021-09-28

**Authors:** Maria Margarete Karsten, Friedrich Kühn, Therese Pross, Jens-Uwe Blohmer, Anna Maria Hage, Felix Fischer, Matthias Rose, Ulrike Grittner, Pimrapat Gebert, Julia Ferencz, Luis Pauler, Clara Breidenbach, Christoph Kowalski, on behalf of the PRO B Steering Board, Gregor Matthesius, Jannis Seemann, Jennifer Lenz, Sophia Rocabado, Marlen Du Bois, Lars Straubing

**Affiliations:** 1 Department of Gynecology with Breast Center, Charité – Universitätsmedizin Berlin, corporate member of Freie Universität Berlin and Humboldt-Universität zu Berlin, 10117 Berlin, Germany; 2 Department of Psychosomatic Medicine, Charité – Universitätsmedizin Berlin, corporate member of Freie Universität Berlin and Humboldt-Universität zu Berlin, Berlin, Germany; 3 Berlin Institute of Health at Charité – Universitätsmedizin Berlin, Berlin, Germany; 4 Institute of Biometry and Clinical Epidemiology, Charité – Universitätsmedizin Berlin, corporate member of Freie Universität Berlin and Humboldt-Universität zu Berlin, Berlin, Germany; 5 OnkoZert GmbH, Neu-Ulm, Germany; 6 Deutsche Krebsgesellschaft e.V., Berlin, Germany

**Keywords:** Metastatic breast cancer, Patient-reported outcomes, Value-based health care, Personalised medicine, Quality of life, Health apps, ePROs

## Abstract

**Background:**

Despite the progress of research and treatment for breast cancer, still up to 30% of the patients afflicted will develop distant disease. Elongation of survival and maintaining the quality of life (QoL) become pivotal issues guiding the treatment decisions. One possible approach to optimise survival and QoL is the use of patient-reported outcomes (PROs) to timely identify acute disease-related burden. We present the protocol of a trial that investigates the effect of real-time PRO data captured with electronic mobile devices on QoL in female breast cancer patients with metastatic disease.

**Methods:**

This study is a randomised, controlled trial with 1:1 randomisation between two arms. A total of 1000 patients will be recruited in 40 selected breast cancer centres. Patients in the intervention arm receive a weekly request via an app to complete the PRO survey. Symptoms will be assessed by study-specific optimised short forms based on the EORTC QLQ-C30 domains using items from the EORTC CAT item banks. In case of deteriorating PRO scores, an alarm is sent to the treating study centre as well as to the PRO B study office. Following the alarm, the treating breast cancer centre is required to contact the patient to inquire about the reported symptoms and to intervene, if necessary. The intervention is not specified and depends on the clinical need determined by the treating physician. Patients in the control arm are prompted by the app every 3 months to participate in the PRO survey, but their response will not trigger an alarm. The primary outcome is the fatigue level 6 months after enrolment. Secondary endpoints include among others hospitalisations, use of rescue services and overall QoL.

**Discussion:**

Within the PRO B intervention group, we expect lower fatigue levels 6 months after intervention start, higher levels of QoL, less unplanned hospitalisations and less emergency room visits compared to controls. In case of positive results, our approach would allow a fast and easy transfer into clinical practice due to the use of the already nationwide existing IT infrastructure of the German Cancer Society and the independent certification institute OnkoZert.

**Trial registration:**

DRKS (German Clinical Trials Register) DRKS00024015. Registered on 15 February 2021

**Supplementary Information:**

The online version contains supplementary material available at 10.1186/s13063-021-05642-6.

## Background

Breast cancer is the most common malignant disease in women worldwide. Despite the progress of research and treatment, up to 30% of the patients afflicted by breast cancer will still develop metastases [1]. With the occurrence of metastases, not only the patients’ life circumstances but also therapeutic approaches change substantially as metastatic breast cancer (MBC) is not yet curable. Prolonging survival and maintaining the best possible quality of life (QoL) by controlling cancer progression and minimising side effects become the leading treatment goals. Fortunately, the overall survival has increased over the last 20 years due to improved treatment options. While the median overall survival was 21 months in 1990, it has been rising steadily to 38 months in 2010 [2]. With an average survival of 38 months and 18,570 annual deaths [3], it can be assumed that currently, more than 60,000 women affected by MBC are living in Germany. The appropriate treatment of these women is still a challenge from a medical, psychosocial and economical point of view. While the disease progresses, the probability of a therapeutic response decreases with each further treatment—it can be less than 15% among previously treated patients [4]. Enormous psychological strain and emotional burden on the patient require extensive support and preventive measures [5]. Furthermore, the treatment costs of advanced breast cancer are currently estimated at about 100,000 US dollars per patient, per year [6, 7]. The aforementioned complexity is a high burden for patients and their treating health care providers and showcases the need to develop new approaches of health care delivery.

One possible approach to optimise overall survival and quality of life is the use of patient-reported outcomes (PROs). Physicians often underestimate the challenge of assessing the severity of symptoms or therapy side effects [8–10]. This particularly concerns unspecific symptoms such as appetite loss and fatigue, although these might indicate disease progression [11]. Electronic PROs deliver real-time data about the patients’ general condition without interpretation by an intermediate authority (i.e. the treating physician) [12] and may detect disease progression and therapy side effects, respectively, at an early stage. Acute disease-related burden can thus be identified with low personnel effort by using mobile devices. In a landmark monocentric study involving 766 patients, Basch et al. were able to show that an intensified PRO monitoring of metastatic cancer patients can lead to an improvement in QoL and to a reduction of unplanned hospitalisations and emergency room visits [13]. Furthermore, it also led to a statistically significant increase in survival [14]. Similar effects regarding the positive impact of an intensified PRO monitoring on survival were shown in a study by Denis et al. increasing the overall survival from 13.5 to 22.5 months in the monitoring group [15]. Müller et al. showed that disease progression is associated with deterioration of health-related quality of life (HRQoL) in patients with MBC [16]. Therefore, early detection of a decrease in HRQoL, which possibly indicates tumour progression, might spare patients treatment cycles with a no longer effective therapy.

With PRO B, we want to investigate whether the benefits of PRO monitoring are transferable from a monocentric study setting into German routine care and if the above-discussed findings can also be shown in breast cancer patients. Therefore, the structure of the project is designed to include all types of care providers in Germany. We will recruit up to 40 breast cancer centres at hospitals in rural and urban areas as well as university hospitals. To facilitate implementation, we will use the existing structures within the network of breast cancer centres certified by the Deutsche Krebsgesellschaft e.V. (DKG, German Cancer Society). Currently, 80.3% of all newly diagnosed breast cancer patients in Germany are treated at a breast cancer centre certified by DKG [17]. In order to obtain certification, every centre has to report structural, processual and outcome data which are audited annually. During PRO B, the data transfer will be conducted by OnkoZert’s IT-tool OncoBox. OncoBox, as implemented in the electronic case report form (PRO B Doc), will enable the connection of clinical and PRO data through an interface of the participating breast cancer centres’ study-specific documentation and a connected smartphone application for the PRO collection.

To our knowledge, this is the first multicentre randomised controlled trial to investigate the effects of PRO in clinical practice on HRQoL and survival exclusively among MBC patients.

## Methods

### Aim

The aim of the PRO B study is to investigate the effects of an electronic PRO monitoring on fatigue (primary outcome), hospitalisations and use of rescue services, physical functioning, overall survival in patients with visceral metastasis, overall survival in patients with triple-negative breast cancer, progression-free survival, health-related quality of life and number of therapy changes (secondary outcomes) among MBC patients in German routine care. The study does not test a specific therapeutic intervention for MBC patients, but rather the impact of an intensified PRO monitoring leading to patient contact in a defined time corridor and possible individual therapy adjustments in the instance of deteriorating PRO values.

### Study design

**Fig. 1:**
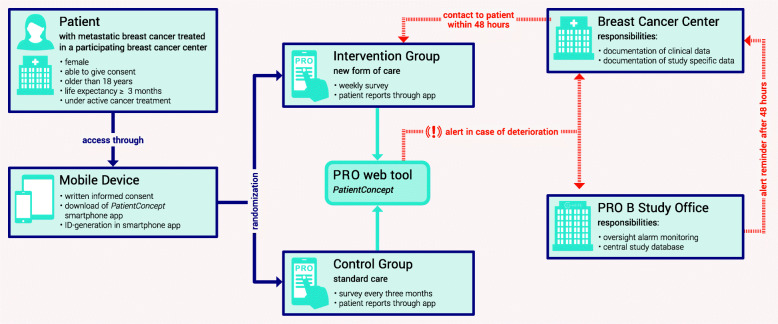
Study design

This study is a randomised controlled, non-blinded superiority trial with 1:1 randomisation between intervention and control. The study design of PRO B is illustrated in Fig. 1. It is based on the earlier cited PRO trials but adapted to the specific conditions of breast cancer care and data protection in Germany. After giving written informed consent and downloading the PRO smartphone application, patients will be randomised. Through the central PRO web tool, the study office allocates the patients to the assigned treatment group by initiating either a weekly or quarterly PRO survey. Among the patients who receive PRO surveys on a weekly basis, the application will generate an alert in the case of worsening PRO values. This alarm will be sent to the breast cancer centre treating the patient as well as to the central study office. Following the alarm, the study sites will contact the patient by phone within 48 h and document the results of the conversation in the web tool. It is up to the treating physicians to decide whether the symptoms the patient describes during the phone contact warrant further interventions and if therapy adjustment has to be applied. If the study centres do not respond to the alert in time, they will receive a reminder from the study office. Neither participants and care providers nor outcome assessors and data analysts can be blinded. This is because the group allocation is traceable on the basis of the number of completed questionnaires and the alerts, which are only generated in the intervention group, at any time.

#### Participants

Patients are eligible if they are female, older than 18 years, able to read and understand German, receive drug treatment for MBC with a life expectancy at enrolment of more than 3 months, if they are insured with one of the three participating health insurance companies and treated in a participating breast cancer centre. Moreover, access to the Internet through a smartphone or tablet, Eastern Cooperative Oncology Group (ECOG) performance status of 0 to 2 and the willingness to participate in a weekly, online-based PRO survey are required.

Patients who do not receive active cancer treatment (comfort care) or do not meet all the above inclusion criteria are not eligible for PRO B.

#### Randomisation

Patients will be randomised to the study group in a 1:1 ratio, and stratified randomisation will be applied according to the following strata:

- Breast cancer centres
- Types of remote metastases (3 groups: bone metastases/skin metastases/lymph node metastases only, visceral metastases (one organ), brain metastases/multiple metastatic sites)
- Histologic findings (2 groups: [HR+/HER−, HR+/HER+], [HR−/HER+, HR−/HER−]).

In order to reduce possible imbalances between the intervention and control groups due to the high number of strata, adaptive randomisation will be performed by the study office using the electronic randomisation software secuTrial® administered by the Clinical Study Centre (CSC) of Charité – Universitätsmedizin Berlin. Randomisation will occur after providing written informed consent, downloading of the application and before the app-based baseline survey (including sociodemographic information and medical history). Group allocation by secuTrial® will be automatically determined for individual patients without a predefined sequence after checking the inclusion criteria, providing a consent form and entering the patient’s characteristics of strata variables by the study team.

#### Intervention group

Patients in the intervention group receive a weekly request (always on the same day of the week) via the app to complete the PRO survey. Analogous to the target parameters of the study, our intervention concept is based on the operationalisation of HRQoL by the European Organisation of Research and Treatment of Cancer (EORTC) Quality of Life Group. Its core instrument, the EORTC QLQ-C30, comprises five functional dimensions (physical function, role function, emotional function, cognitive function, social function) and nine symptom scales (fatigue, nausea, pain, dyspnoea, insomnia, appetite loss, constipation, diarrhoea, financial difficulties) [18]. Those domains will be assessed by questionnaires specifically optimised for the study with items from the respective EORTC item bank [19]. We used Item Response Theory to construct short forms of varying length and item content for each domain, tailored to achieve maximum precision in the expected range of scores. For the health economic evaluation, the questionnaire EQ-5D will be used [20]. All PRO surveys will be administered in German.

In the case of clinically relevant deterioration of PRO scores regarding the individual longitudinal course, an alarm is generated. Values will be automatically compared to the scores of the previous week for short-term changes and to the mean of the scores 3, 4 and 5 weeks before in order to detect long-term changes of the HRQoL.

#### Control group

Patients in the control arm are prompted by the app every 3 months to participate in the PRO survey. For the control group, there is no alert in case of deterioration of the PRO values and thus no contact. In case of non-response to the survey, a reminder is sent by the study centre.

In order to keep the dropout rate in the control group as low as possible, patients will receive a printout or file of their survey data, i.e. their PRO history, after completion of the observation period. According to the feedback of the breast cancer self-aid group at Charité – Universitätsmedizin Berlin, the intensified digital monitoring of the PRO course is considered a sufficient incentive for the intervention group.

#### Adverse events

No adverse events as a consequence of a smartphone-based digital PRO monitoring are expected. Since only patients who are willing to participate in a regular PRO survey after being informed about the extent and content of the questionnaires are eligible for the study, we do not anticipate adverse events or sensitivity towards these. Consequently, no provisions for ancillary and post-trial care and for compensation for harm from participation are taken.

#### Sample size/recruitment

In a meta-analysis of studies concerning changes in the scores of EORTC’s QLQ-C30, Cocks et al. analysed 35 studies reporting improvement on the C30 fatigue scale [21]. The mean improvement is reported as an increase of 5 points. The authors developed guidelines for interpreting longitudinal changes of the QLQ-C30 fatigue scale defining an increase of 4 points as minor and an increase of 9 points as a moderate improvement. We have based our effect estimates on that information. However, the project investigates the differences between a treatment and a control group, which lets us expect greater measures of dispersion. Conservatively estimated, we therefore expect a standardised effect size of 0.2 (e.g. mean difference of 5 points, standard deviation 25.2 points). These values are comparable to those of Cortes et al. [22].

Since the analysis of certain domains is of high relevance in this project, we strive for a case number high enough for subset analyses. The recruitment of 500 patients per study arm is considered realistic. We expect a dropout rate of about 20%, which would result in approximately 400 complete datasets per treatment arm. Based on the earlier cited investigation of Cocks et al. reporting a minimal clinically relevant effect on the EORTC QLQ-C30 fatigue scale of five points mean difference (with a joint standard deviation of 25), a two-sample *t*-test with a significance level of 5% would have a power of 80.65%. Thus, the case number would be sufficient to detect small effect sizes (≥ 0.2) while taking into account the number of relevant subgroups in the patient population. This calculation was conducted with nQuery (version 8.3.1.0) [23].

A thousand participants will be recruited in up to 40 certified breast cancer centres (municipal and district hospitals, university hospitals) throughout Germany starting in May 2021. Potential study centres were approached first by the number of patients insured with the cooperating health insurance companies in the previous year and second by the number of overall cases in the previous years to increase chances of sufficiently large sample sizes per hospital.

#### Primary hypothesis

Patients receiving weekly PRO surveys (intervention group) will have a statistically significantly lower fatigue level after 6 months compared to patients receiving only quarterly PRO surveys (control group).

#### Secondary hypotheses

Compared to the control group, patients in the intervention group will:

- Need fewer hospital stays and emergency room visits after 6 and 12 months
- Have a higher physical functioning level after 6 and 12 months
- Have a lower fatigue level after 12 months
- Have a higher survival rate in cases of visceral metastases after 12 months
- Have a higher survival rate in cases of triple-negative breast cancer after 12 months
- Have an increase in progression-free survival after 12 months
- Have a higher sum score of HRQoL after 6 and 12 months
- Have a higher number of therapy changes due to early detection of disease progression

### Statistical analysis

#### Primary outcome

The primary research question will be examined in an intention-to-treat analysis for all randomised individuals. All stratification variables of randomisation are included as covariates in all statistical models.

We used a two-sample *t*-test for the sample size calculation although the final evaluation will be performed using an ANCOVA model. Assuming *ρ* (rho) as the variance inflation factor (correlation between baseline and follow-up measurement), it has been shown that this is a conservative approach for sample size estimations of ANCOVA models, since an ANCOVA model with (1 − 2*ρ*)×*n* observations has at least the same power as a *t*-test with *n* observations [24]. In the worst case of *ρ* = 0, a sample size equal to the one of the *t*-test is required. The analysis will be performed in the ANCOVA model with baseline measurement and type of metastases/histology as covariates. In addition, the analysis will be adjusted for age, type and number of system therapies, ECOG status at enrolment, smartphone experience and other important confounders if necessary. The heterogeneity between the trial sites is considered through random effects (random intercept mixed model).

#### Secondary outcomes

All secondary hypotheses will be analysed analogously to the primary hypothesis and according to the respective outcome using suitable statistical methods (e.g. time-to-event models, ANCOVA, logistic regression, Cox regression). As in the analysis of the primary outcome, the secondary hypotheses will be adjusted for relevant confounders. Also, the heterogeneity between study centres will be considered.

All statistical analyses will be planned in detail and documented in a statistical analysis plan (SAP) that will be prepared before the closure of the database.

#### Interim analyses

This protocol does not foresee any interim analyses. We expect the impact of the digital PRO-monitoring on our primary endpoint to occur only after a certain amount of time. According to the above-mentioned pilot studies, we assume 6 months post-enrolment to be suitable to detect early effects. Sufficient statistical power and health economic analyses will only be possible within the final evaluation at the end of the study. Against this backdrop, no guidelines concerning premature stopping have been defined. In the case of any unforeseeable events requiring immediate closure of the study, the project leader makes the final decision to stop the trial.

#### Data acquisition and management

We will capture PRO data (including the primary endpoint fatigue) on a weekly or quarterly basis using a smartphone application and thereby ensuring standardised data acquisition. Data will be collected in a central study database monitored by an external clinical research organisation (CRO) which will also conduct regular audits regarding protocol compliance. To ensure that physicians from attending study sites contact their patients in case of worsening PRO values, we will integrate an alert system, which informs the respective physician/study nurse as well as the PRO B study office. In the event of a belated or omitted response (e.g. > 48 h), the study office will send a reminder to the participating site.

**Fig. 2:**
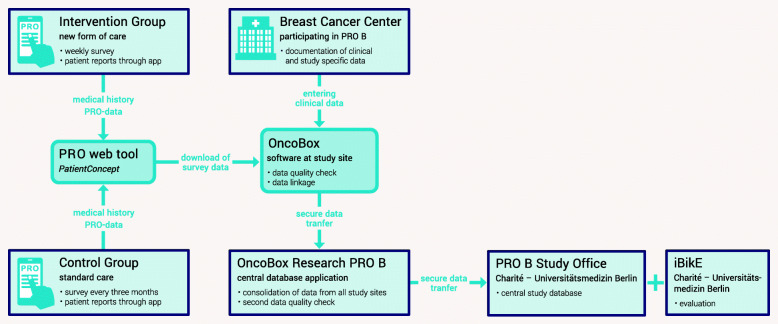
Data flow

In certified German cancer centres, standardised clinical tumour documentation systems are widely established mostly because they are used in the certification processes of the German Cancer Society [25]. For PRO B, the existing IT infrastructure will be extended by further items in a new application (PRO B Doc). The PRO survey tool is established and in use for other medical conditions and will be adapted for the project [26]. Both clinical and PRO data will be collected and then matched in the central database (Fig. 2). Automated checks on plausibility and additional onsite visits for source data reconciliation ensure a high quality of the final dataset. We cooperate with three major German statutory health insurance companies, which will supply secondary data at the end of the study for the economic analyses. The integration of these data will be handled by our institutional trust centre and data integration centre. The evaluation of the data is performed by the Institute of Biometry and Clinical Epidemiology (iBiKE) of the Charité – Universitätsmedizin Berlin. We do not consider a data monitoring committee (DMC) necessary as the participating party OnkoZert and the Charité Clinical Study Centre (= CRO) as well as the health insurance companies provide very experienced data managers.

#### Analysis of subgroups

All statistical subset analyses are explorative. In order to analyse the first indications for differential treatment effects in the subgroups, an additional interaction term of the treatment variable will be included with the subgroup in the ANCOVA model (primary or secondary outcome, covariables: respective baseline variable, treatment variable, stratification variable of randomisation, random intercept for the study sites). We will report marginal effect estimators for each subgroup and 95% confidence intervals.

#### Health economic evaluation

Total treatment costs for each patient are captured and analysed considering the individual observation period (average treatment costs per month per patient). Taking into account the skewed distribution of the costs, we use generalised linear models with gamma distribution and log link function for statistical analysis or data transformation if necessary. Further statistical methods for economic analyses will be specified in the statistical analysis plan during the project. Cost efficacy analyses will be conducted if the intervention shows to be superior concerning “quality-adjusted life years” (QALYs) which consider both lifetime and QoL. They are calculated based on QoL questionnaires (EQ-5D) answered at baseline and various follow-up time points, assuming a linear temporal change in QoL between the measurement points. QALYs result from the calculation of the area under the curve. The intervention is considered cost-effective if it leads to QoL improvement at lower costs. If the intervention causes additional costs, we will calculate the incremental cost-effectiveness ratio (ICER) by estimating the mean additional costs per additional QALY. In studies, a limit of €50,000 per extra QALY is often used as a threshold for cost-effectiveness of an intervention. With a lower ICER, the intervention is considered cost-effective.

#### Dropouts and missing data

Reasons for dropouts and any case of missing values will be documented and reported as far as possible. Assuming that missing values are “missing at random” (MAR), they will be imputed using multiple imputation methods. Sensitivity analyses will be conducted and used for examining the assumptions of missing data mechanisms.

#### Dissemination

Any protocol modifications will be submitted for approval to the research ethics committee and disseminated by e-mail to site principal investigators and trial coordinators. The statistician and health economists will have access to the pseudonymised final linked trial dataset. There are no plans to provide public access to the full protocol, participant-level data or statistical code. The researchers aim to publish results in a peer-reviewed journal and share them via social media and conferences. Authorship guidelines according to the International Committee of Medical Journal Editors (ICMJE) will be applied.

## Discussion

Within the PRO B intervention group, we expect the QoL to be improved and unplanned hospitalisations, emergency room visits as well as treatment costs to be reduced through early detection of decreasing QoL.

In case of positive results, our approach would allow a fast and easy transfer into clinical practice due to the use of the already nationwide existing IT infrastructure of the German Cancer Society (DKG) and OnkoZert.

We recently implemented electronic PROs in the adjuvant setting at our own institution and recognised a high acceptance and response rate among all age groups [27]. Up until now, PRO measurement is not standardised and did not yet reach clinical practice throughout the country. With PRO B, a first step towards a nationwide, comprehensive, standardised, affordable and easy collection of PRO data will be done, possibly enabling a better assessment of the consequences of breast cancer treatment and its related implications on the patients’ quality of life. This information could be of great value in the counselling and decision-making processes for both practitioners and patients with metastatic breast cancer.

## Trial status

Recruitment started on 17 May 2021. The end of recruitment is planned for May 2022. The end of the follow-up period is planned for May 2023. First, results are expected in September 2023. The current paper describes protocol version 2.2 (2 April 2021) and was prepared according to the SPIRIT checklist (Additional file 2).

## Supplementary Information

**Additional file 1.** Consent form for patients treated at Charité - Universitätsmedizin Berlin (German).

**Additional file 2.** SPIRIT 2013 Checklist: Recommended items to address in a clinical trial protocol and related documents*.

## Data Availability

Data sharing is not applicable to this article as no datasets were generated or analysed.

## Abbreviations

CRO: Clinical Research Organisation
CSC: Clinical Study Centre
DKG: German Cancer Society (Deutsche Krebsgesellschaft)
ECOG: Eastern Cooperative Oncology Group
EORTC: European Organisation for Research and Treatment of Cancer
GCP: Good Clinical Practice
HER: Human epidermal growth factor receptor 2
HR: Hormone receptor
HRQoL: Health-related quality of life
iBiKE: Institute of Biometry and Clinical Epidemiology (Institut für Biometrie und Klinische Epidemiologie)
ICER: Incremental cost-effectiveness ratio
ICMJE: International Committee of Medical Journal Editors
MAR: Missing at random
MBC: Metastatic breast cancer
PRO: Patient-reported outcome
QALY: Quality-adjusted life year
QoL: Quality of life
SAP: Statistical analysis plan
