# Supplementary material for: PRO B: evaluating the effect of an alarm-based patient-reported outcome monitoring compared with usual care in metastatic breast cancer patients—study protocol for a randomised controlled trial
Source: Trials. 2021 Sep 28;22:666. doi: 10.1186/s13063-021-05642-6 (PMC8479993; doi:10.1186/s13063-021-05642-6)
Supplement: Supplementary file 1 — Additional file 1. Consent form for patients treated at Charité - Universitätsmedizin Berlin (German). [file 13063_2021_5642_MOESM1_ESM.pdf]

Charité | Campus Charité Mitte | 10117 Berlin

Klinik für Gynäkologie mit Brustzentrum

PRO B

Dr. med. Jenny Katharina Wagner | Studienärztin

Post: Charitéplatz 1
10117 Berlin
Tel.: +49 (0)30 450 664 426
E-Mail: jenny-katharina.wagner@charite.de

Prof. Dr. med. Jens-Uwe Blohmer | Klinikdirektor

Homepage Frauenklinik: http://frauenklinik.charite.de

Einwilligungserklärung

für die Nutzung von Patientinnen- und Krankenkassendaten für das Forschungsprojekt „PRO B – Neue Wege in der Versorgung bei metastasiertem Brustkrebs“

Nachname, Name: ______________________________

Straße, Hausnummer: ______________________________

PLZ, Wohnort: ______________________________

Telefon: ______________________________

E-Mail: ______________________________

Krankenkasse und Krankenversichertennummer: ______________________________

Hiermit erkläre ich, dass ich durch Herrn*Frau

....
*(Name des*der aufklärenden Arztes/Ärztin)*

mündlich und schriftlich über das Wesen, die Bedeutung und die Risiken der wissenschaftlichen Untersuchungen im Rahmen der Studie „PRO B – Neue Wege in der Versorgung bei metastasiertem Brustkrebs“ informiert wurde und ausreichend Gelegenheit hatte, meine Fragen zu klären.
Mir ist bekannt, dass bei einer Teilnahme an dem Forschungsprojekt PRO B umfangreiche personenbezogene Daten, insbesondere auch sensible Daten zu meiner Gesundheit Daten zu meinen

wirtschaftlichen und familiären Verhältnissen sowie Daten der Krankenkasse zu meiner ambulanten Versorgung in pseudonymisierter Form verarbeitet und ausgewertet werden sollen. Daten zu meiner Gesundheit und meiner Lebensqualität sollen bei Teilnahme regelmäßig, bis zu einmal wöchentlich, über eine App bzw. ein Web-Portal abgefragt werden. Mir ist bekannt, dass ich jederzeit die Auskunft (inklusive unentgeltliche Kopie der mich betreffenden Daten nach Art. 15 DS-GVO) meiner Daten verlangen kann.

Ich wurde anhand der *Information für Patientinnen* und *Datenschutzinformation* vom 02.04.2021 ausführlich und verständlich über die Verarbeitung meiner personenbezogenen Daten sowie die damit verbundenen Risiken aufgeklärt. Ich hatte ausreichend Bedenkzeit und alle meine Fragen wurden zufriedenstellend beantwortet.

Ich habe eine Kopie der schriftlichen *Information für Patientinnen*, *Datenschutzinformation* und der Einwilligungserklärung jeweils mit Versionsdatum vom 02.04.2021 erhalten.

<u>Einwilligung</u>

Ich erkläre mich freiwillig bereit, an der wissenschaftlichen Studie "PRO B – Neue Wege in der Versorgung bei metastasiertem Brustkrebs" teilzunehmen.

1. **Ich willige ein in die Erhebung, Verarbeitung, Speicherung und Auswertung meiner personenbezogenen Daten für wissenschaftliche Zwecke in Zusammenhang mit der Studie, wie es in der *Information für Patientinnen* und *Datenschutzinformation* beschrieben ist, einschließlich in**

    - **die Erhebung meiner Patientinnendaten, Daten zu meinen soziodemographischen und sozioökonomischen Umständen, meinem Gesundheitszustand und meiner Lebensqualität, jeweils in dem für die Durchführung der Studie erforderlichen Umfang;**
    - **die Verarbeitung dieser Daten in pseudonymer Form, d.h. getrennt von meinen direkt identifizierenden Stammdaten und mithilfe von Codierungen;**
    - **die Zusammenführung dieser Daten mit meinen von der Krankenkasse übermittelten weiteren Daten in Datenbanken der Charité – Universitätsmedizin Berlin (Klinik für Gynäkologie mit Brustzentrum sowie Institut für Biometrie und Klinische Epidemiologie), Charitéplatz 1, 10117 Berlin;**
    - **die wissenschaftliche Auswertung dieser Daten und die anschließende Speicherung für zehn Jahre zu Nachweiszwecken;**
    - **die Anonymisierung dieser Daten, sodass eine Zuordnung zu meiner Person ausgeschlossen ist, etwa für Zwecke der anschließenden Veröffentlichung der Studienergebnisse oder der anschließenden Weitergabe der Daten an Kooperationspartner*innen bzw. wissenschaftliche Fachzeitschriften;**
    - **die Verarbeitung meiner Stammdaten zu Zwecken der Kontaktaufnahme und Abstimmung der weiteren Maßnahmen durch behandelnde Studienärzte*-ärztinnen des Brustkrebszentrums im Falle einer Verschlechterung der Werte;**
    - **die Einsicht in die Originalunterlagen durch die Studienmonitor*innen bzw. Auditor*innen der CRO (Charité-BIH Clinical Study Center) im Rahmen des Studienmonitorings und der Quelldatenabgleiche, sowie ggf. bei Behördeninspektionen und entbinde hierfür die behandelnden Ärzt*innen von ihrer Schweigepflicht.**

2. **Ich ermächtige meine Krankenkasse, auf Anforderung durch die Datentreuhandstelle der Charité – Universitätsmedizin Berlin Daten über von mir in Anspruch genommene ärztliche Leistungen in der ambulanten Versorgung und bei stationären Aufenthalten, über verordnete Heil- und Hilfsmittel sowie Arzneimittel und Angaben zum Bereich Pflege an das Datenintegrationszentrum der Charité – Universitätsmedizin Berlin zu übermitteln. Dies soll für Daten ab dem Datum meiner Unterschrift und für einen Zeitraum von 36 Monaten gelten.**

   Ich willige ein in

   - **die hierfür erforderliche Übermittlung meiner Krankenversicherungs-Nummer und**
   - **die weitere Verarbeitung der Krankenkassendaten in pseudonymer Form für wissenschaftliche Zwecke in Zusammenhang mit der Studie PRO B, wie es vorstehend, in der *Information für Patientinnen* und in der *Datenschutzinformation* beschrieben ist.**

3. ***Nur für Patientinnen der Charité:* Ich willige darin ein, dass dem beim Charité Comprehensive Cancer Center geführten klinischen Krebsregister meine Teilnahme an dieser onkologischen Studie personenbezogen zum Zweck der Qualitätssicherung der Tumorbehandlung gemeldet wird.**

Widerrufsrecht

Diese Einwilligung ist freiwillig. Ich kann meine Einwilligung jederzeit ohne Angabe von Gründen mit Wirkung für die Zukunft widerrufen (z.B. in Textform per Brief oder E-Mail), ohne dass mir irgendwelche Nachteile entstehen.

Beim Widerruf werden die auf Grundlage dieser Einwilligung gespeicherten Daten gelöscht oder anonymisiert, sofern keine gesetzlichen Gründe entgegenstehen. Mir ist zudem bekannt, dass Daten, die bereits in wissenschaftliche Auswertungen eingeflossen sind, nicht mehr nachträglich entfernt werden können.

Berlin, den ______________________________

____

Unterschrift der Teilnehmerin

Hiermit erkläre ich, dass ich die Teilnehmerin am ……………………...... über Wesen, Bedeutung und Risiken der o. g. Studie mündlich und schriftlich aufgeklärt, alle Fragen beantwortet und ihr eine Kopie der Studieninformation, Datenschutzinformation und der Einwilligungserklärung übergeben habe.

Berlin, den ______________________________

____

Name und Unterschrift des*der aufklärenden Arztes/Ärztin
