# Supplementary material for: PRO B: evaluating the effect of an alarm-based patient-reported outcome monitoring compared with usual care in metastatic breast cancer patients—study protocol for a randomised controlled trial
Source: Trials. 2021 Sep 28;22:666. doi: 10.1186/s13063-021-05642-6 (PMC8479993; doi:10.1186/s13063-021-05642-6)
Supplement: Supplementary file 2 — Additional file 2. SPIRIT 2013 Checklist: Recommended items to address in a clinical trial protocol and related documents*. [file 13063_2021_5642_MOESM2_ESM.docx]

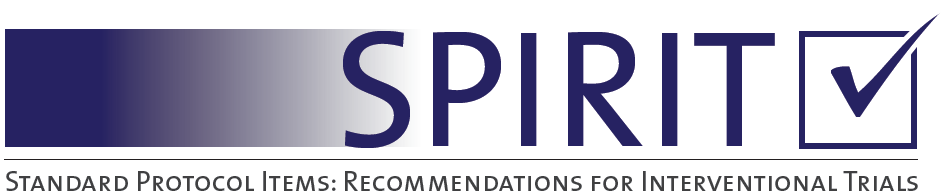


SPIRIT 2013 Checklist: Recommended items to address in a clinical trial protocol and related documents*

| Section/item | Item No | Description | Addressed on page number |
| --- | --- | --- | --- |
| **Administrative information** | | |  |
| Title | 1 | Descriptive title identifying the study design, population, interventions, and, if applicable, trial acronym | p. 1 / Title page |
| Trial registration | 2a | Trial identifier and registry name. If not yet registered, name of intended registry | p. 2 / Abstract paragraph 4; p. 14 / Trial registration |
|  | 2b | All items from the World Health Organization Trial Registration Data Set | The trial has been registered in the German Clinical Trials Register (DRKS) which collects all items from the World Health Organization Trial Registration Data Set. (p. 2 / Abstract, paragraph 4; p. 14 / Trial registration) |
| Protocol version | 3 | Date and version identifier | p. 12 / Trial status |
| Funding | 4 | Sources and types of financial, material, and other support | p. 13 / Funding |
| Roles and responsibilities | 5a | Names, affiliations, and roles of protocol contributors | p. 1 / Title page; p. 12 / Authors’ contributions |
|  | 5b | Name and contact information for the trial sponsor | n. a. (According to German law, a sponsor is required for studies pursuant to the German Medicines Act (AMG, §4 para. 24) or the German Medical Devices Act (MPG, §3, para. 23). PRO B is a non-AMG / non-MPG trial which is why determining a sponsor is not mandatory.) |
|  | 5c | Role of study sponsor and funders, if any, in study design; collection, management, analysis, and interpretation of data; writing of the report; and the decision to submit the report for publication, including whether they will have ultimate authority over any of these activities | p. 13 / Funding |
|  | 5d | Composition, roles, and responsibilities of the coordinating centre, steering committee, endpoint adjudication committee, data management team, and other individuals or groups overseeing the trial, if applicable (see Item 21a for data monitoring committee) | The steering board of PRO B consists of the authors of the protocol affiliated with Charité, German Cancer Society, OnkoZert and the three health insurance companies, whose contributions are described on p. 13-14 and throughout the manuscript. |
| Introduction |  |  |  |
| Background and rationale | 6a | Description of research question and justification for undertaking the trial, including summary of relevant studies (published and unpublished) examining benefits and harms for each intervention | p. 3-4 / Background, paragraph 1-4 |
|  | 6b | Explanation for choice of comparators | n.a. (This applies to drug studies. PRO B evaluates the effect of a new form of care (intensified digital PRO monitoring) compared to usual / standard care of metastatic breast cancer patients) |
| Objectives | 7 | Specific objectives or hypotheses | p. 8 / Methods, “Primary hypothesis” and “Secondary hypotheses” |
| Trial design | 8 | Description of trial design including type of trial (eg, parallel group, crossover, factorial, single group), allocation ratio, and framework (eg, superiority, equivalence, noninferiority, exploratory) | p. 5 / Methods “Study design”, paragraph 1 |
| Methods: Participants, interventions, and outcomes | | |  |
| Study setting | 9 | Description of study settings (eg, community clinic, academic hospital) and list of countries where data will be collected. Reference to where list of study sites can be obtained | p. 8 / Methods “Sample size / Recruitment”, paragraph 3 / p. 14 / Acknowledgements (Website with list of study sites) |
| Eligibility criteria | 10 | Inclusion and exclusion criteria for participants. If applicable, eligibility criteria for study centres and individuals who will perform the interventions (eg, surgeons, psychotherapists) | p. 5-6 / Methods “Participants”, paragraph 1 and 2 |
| Interventions | 11a | Interventions for each group with sufficient detail to allow replication, including how and when they will be administered | p. 6-7 / Methods “Intervention group” and “Control group” |
|  | 11b | Criteria for discontinuing or modifying allocated interventions for a given trial participant (eg, drug dose change in response to harms, participant request, or improving/worsening disease) | n.a. (Except for withdrawal of consent and death, which are taken for granted, no additional criteria for discontinuing the intervention have been defined.) |
|  | 11c | Strategies to improve adherence to intervention protocols, and any procedures for monitoring adherence (eg, drug tablet return, laboratory tests) | p. 7 / Methods “Control Group”, paragraph 2 |
|  | 11d | Relevant concomitant care and interventions that are permitted or prohibited during the trial | n.a. (PRO B aims to establish an intensified digital PRO monitoring as part of the care of metastatic breast cancer patients independent from their individual treatment. Any kind of concomitant care and other interventions are therefore permitted. Patients who receive comfort care in the first place are not eligible (p. 6 / paragraph 1) |
| Outcomes | 12 | Primary, secondary, and other outcomes, including the specific measurement variable (eg, systolic blood pressure), analysis metric (eg, change from baseline, final value, time to event), method of aggregation (eg, median, proportion), and time point for each outcome. Explanation of the clinical relevance of chosen efficacy and harm outcomes is strongly recommended | 5-7 / Methods “Aim”, “Intervention Group” |
| Participant timeline | 13 | Time schedule of enrolment, interventions (including any run-ins and washouts), assessments, and visits for participants. A schematic diagram is highly recommended (see Figure) | p. 12 / Trial Status (PRO B does not foresee any specific visits for participants. It offers an accompanying digital monitoring of the health status and quality of life during their treatment alerting the treating physician in case of deterioration. Patients in the intervention group receive a weekly reminder to answer a PRO survey on their smartphone – the protocol does not prescribe the measures, which might follow an alert as the study is intended to evaluate a timely response to worsening PRO scores rather than specific measures against that worsening. Against this backdrop, the SPIRIT figure does not seem suitable for this study. The intervention is described on p. 5 and 6-7 / Methods, “Study design” and “Intervention group”) |
| Sample size | 14 | Estimated number of participants needed to achieve study objectives and how it was determined, including clinical and statistical assumptions supporting any sample size calculations | p. 7-8 / Methods “Sample size / Recruitment” |
| Recruitment | 15 | Strategies for achieving adequate participant enrolment to reach target sample size | p. 8 / Methods “Sample size / Recruitment”, paragraph 3 |
| **Methods: Assignment of interventions (for controlled trials)** | | |  |
| Allocation: |  |  |  |
| Sequence generation | 16a | Method of generating the allocation sequence (eg, computer-generated random numbers), and list of any factors for stratification. To reduce predictability of a random sequence, details of any planned restriction (eg, blocking) should be provided in a separate document that is unavailable to those who enrol participants or assign interventions | p. 6 / Methods “Randomisation” (Randomisation will be conducted using a randomisation tool embedded in the secuTrial® software administered by the involved clinical research organisation.) |
| Allocation concealment mechanism | 16b | Mechanism of implementing the allocation sequence (eg, central telephone; sequentially numbered, opaque, sealed envelopes), describing any steps to conceal the sequence until interventions are assigned | p. 6 / Methods “Randomisation” |
| Implementation | 16c | Who will generate the allocation sequence, who will enrol participants, and who will assign participants to interventions | p. 5 / 6 / Methods “Study Design” and “Randomisation” (The randomisation is automated as embedded in the secuTrial® software administered by involved the clinical research organisation.) |
| Blinding (masking) | 17a | Who will be blinded after assignment to interventions (eg, trial participants, care providers, outcome assessors, data analysts), and how | p. 5 / study design |
|  | 17b | If blinded, circumstances under which unblinding is permissible, and procedure for revealing a participant’s allocated intervention during the trial | p. 5 / study design (PRO B is a non-blinded RCT. Neither participants and care providers nor outcome assessors and data analysts can be blinded. This is because the group allocation is traceable on the basis of the number of completed questionnaires and the alerts (which are only generated in the intervention group) at any time.) |
| **Methods: Data collection, management, and analysis** | | |  |
| Data collection methods | 18a | Plans for assessment and collection of outcome, baseline, and other trial data, including any related processes to promote data quality (eg, duplicate measurements, training of assessors) and a description of study instruments (eg, questionnaires, laboratory tests) along with their reliability and validity, if known. Reference to where data collection forms can be found, if not in the protocol | p.6-7; / Methods “Intervention Group”, p. 10 / Data acquisition and managament |
|  | 18b | Plans to promote participant retention and complete follow-up, including list of any outcome data to be collected for participants who discontinue or deviate from intervention protocols | p. 7 / Methods “Control Group” paragraph 2, p. 11 / Methods “Dropouts and missing data” |
| Data management | 19 | Plans for data entry, coding, security, and storage, including any related processes to promote data quality (eg, double data entry; range checks for data values). Reference to where details of data management procedures can be found, if not in the protocol | p. 10 / Methods “Data acquisition and management”, paragraphs 1-2 (PRO B is based on a data protection concept, which is only available in German.) |
| Statistical methods | 20a | Statistical methods for analysing primary and secondary outcomes. Reference to where other details of the statistical analysis plan can be found, if not in the protocol | p. 9-11 / Methods “Statistical analyses” |
|  | 20b | Methods for any additional analyses (eg, subgroup and adjusted analyses) | p. 10 / Methods “Analysis of subgroups” |
|  | 20c | Definition of analysis population relating to protocol non-adherence (eg, as randomised analysis), and any statistical methods to handle missing data (eg, multiple imputation) | p. 11 / Methods “Dropouts and missing data” |
| **Methods: Monitoring** | | |  |
| Data monitoring | 21a | Composition of data monitoring committee (DMC); summary of its role and reporting structure; statement of whether it is independent from the sponsor and competing interests; and reference to where further details about its charter can be found, if not in the protocol. Alternatively, an explanation of why a DMC is not needed | p. 10 / Methods “Data acquisition and management” |
|  | 21b | Description of any interim analyses and stopping guidelines, including who will have access to these interim results and make the final decision to terminate the trial | p. 9 / Methods “Interim analyses” |
| Harms | 22 | Plans for collecting, assessing, reporting, and managing solicited and spontaneously reported adverse events and other unintended effects of trial interventions or trial conduct | p. 7 / Methods “Adverse events” |
| Auditing | 23 | Frequency and procedures for auditing trial conduct, if any, and whether the process will be independent from investigators and the sponsor | p. 10 / Methods “Data acquisition and management”, paragraphs 1-2 |
| Ethics and dissemination | | |  |
| Research ethics approval | 24 | Plans for seeking research ethics committee/institutional review board (REC/IRB) approval | p. 12 / Ethics approval and consent to participate |
| Protocol amendments | 25 | Plans for communicating important protocol modifications (eg, changes to eligibility criteria, outcomes, analyses) to relevant parties (eg, investigators, REC/IRBs, trial participants, trial registries, journals, regulators) | p. 11 / Methods “Dissemination” |
| Consent or assent | 26a | Who will obtain informed consent or assent from potential trial participants or authorised surrogates, and how (see Item 32) | p. 12 / Ethics approval and consent to participate |
|  | 26b | Additional consent provisions for collection and use of participant data and biological specimens in ancillary studies, if applicable | p. 12 / Ethical approval and consent to participate |
| Confidentiality | 27 | How personal information about potential and enrolled participants will be collected, shared, and maintained in order to protect confidentiality before, during, and after the trial | p. 10 / Methods “Data acquisition and management”, p. 11 / “Dissemination” (Additional data protection measures are defined in a separate data protection concept available only in German. PRO B uses already existing IT elements from the certification process of the German Cancer Society, described in the manuscript on p. 4 para. 2 and p. 10 para. 3, and an established and CE-certified App.) |
| Declaration of interests | 28 | Financial and other competing interests for principal investigators for the overall trial and each study site | p. 13 / Competing interests |
| Access to data | 29 | Statement of who will have access to the final trial dataset, and disclosure of contractual agreements that limit such access for investigators | p. 11 / Methods “Dissemination” |
| Ancillary and post-trial care | 30 | Provisions, if any, for ancillary and post-trial care, and for compensation to those who suffer harm from trial participation | p. 7 / Methods “Adverse Events” |
| Dissemination policy | 31a | Plans for investigators and sponsor to communicate trial results to participants, healthcare professionals, the public, and other relevant groups (eg, via publication, reporting in results databases, or other data sharing arrangements), including any publication restrictions | p. 11 / Methods “Dissemination” |
|  | 31b | Authorship eligibility guidelines and any intended use of professional writers | p. 11 / Methods “Dissemination” (There are no plans to use professional writers in the context of that study.) |
|  | 31c | Plans, if any, for granting public access to the full protocol, participant-level dataset, and statistical code | p. 11 / Methods “Dissemination” |
| Appendices |  |  |  |
| Informed consent materials | 32 | Model consent form and other related documentation given to participants and authorised surrogates | p. 12 / Ethical approval and consent to participate (The consent form is only available in German. We submitted it as Supplement 1) |
| Biological specimens | 33 | Plans for collection, laboratory evaluation, and storage of biological specimens for genetic or molecular analysis in the current trial and for future use in ancillary studies, if applicable | p 12 / Ethical approval and consent to participate (No samples will be collected.) |

*It is strongly recommended that this checklist be read in conjunction with the SPIRIT 2013 Explanation & Elaboration for important clarification on the items. Amendments to the protocol should be tracked and dated. The SPIRIT checklist is copyrighted by the SPIRIT Group under the Creative Commons “[Attribution-NonCommercial-NoDerivs 3.0 Unported](http://www.creativecommons.org/licenses/by-nc-nd/3.0/)” license.
